# Gender Differences in Migrant Workers Health in China

**DOI:** 10.3389/ijph.2023.1605018

**Published:** 2023-08-14

**Authors:** Yihao Tian, Yong Zhan, Min Wu

**Affiliations:** Department of Labour and Social Security, School of Public Administration, Sichuan University, Chengdu, China

**Keywords:** gender differences, health inequalities, China, health insurance, education level, migrant workers

## Abstract

**Objectives:** This study aimed to explore the distribution and differences in the health status of migrant workers in China by gender and age. In addition, it investigated the causes of health inequalities among them.

**Methods:** This paper analyzes the differences in health status across age groups for migrant workers of different genders based on the data from the China Migrant Dynamic Survey in 2018. It also empirically assesses how education level and health insurance impact gender-related health inequalities.

**Results:** The results suggest that female migrant workers in China have significantly lower health levels than males. Furthermore, these differences in health are exacerbated with age. This disparity may be attributed to lower participation in social insurance participation and less educational attainment among female migrant workers than their male counterparts.

**Conclusion:** The government should take effective practical measures to increase the social insurance participation rate of female migrant workers. Moreover, investing in female education to reduce health inequality among migrant workers is essential.

## Introduction

Migrants have significantly contributed to the rapid development of China’s economy and the urbanization process [1]. As of 2020, the number of migrants in China has reached 376 million, of whom 48.6% were female. Female migrant workers have successively entered the labor market, making indelible contributions to social and economic development [2]. However, compared with males, female migrant workers experience apparent inequality in employment, housing, medical care, pension, and other areas due to their weaker labor-market attachment, lower socio-economic status, and the restriction of stereotypes [3–7]. Health is a critical issue that people attend to as the guarantee of individual accumulation of human capital and the pursuit of high quality of life. The health level of females is related to their individual situations. However, it also impacts the physical wellbeing of their children, which, in turn, is closely related to family happiness, social stability, and sustainable development. Therefore, investigating the health inequality of migrant workers and exploring its causes will be of great significance in reducing gender inequality and improving the health level of females.

### Literature Review

The root causes of gender differences in health are multifaceted and interrelated. Studies have shown that females have a lower mortality rate, but their overall incidence of physical diseases is higher than that of males [8]. The existing literature mainly explains the phenomenon of health inequalities from three perspectives: socio-economic status, lifestyle, and social psychology. First, regarding socio-economic status, studies have demonstrated the unequal relationship between health inequality and income, education, and occupation [9, 10]. Compared to males, female migrant workers find it more difficult to get leave, have relatively low work income, heavier work and life burdens, and their health level is usually lower than that of male migrant workers [11]. Second, lifestyles (such as exercise, diet, sleep, and smoking) are also closely linked to health [12]. A healthy lifestyle can improve the quality of life for migrant workers. However, female migrant workers face more household jobs, making them less likely to have healthy behavior than their husbands [13, 14]. Finally, life events, chronic stress sources, and psychological resources also affect health (especially for females), as female migrant workers face a higher level of demands and obligations in social roles and are more vulnerable to stress [15, 16].

These studies take into account the impact of socio-economic status, lifestyle, and psycho-social factors on the health status of different gender groups. Yet the literature pays little attention to the existence of gender differences in health status among migrant workers of different ages and their possible causes.

Based on the China Migrant Dynamic Survey (CMDS) from 2018, this paper uses a multiple linear regression model to investigate the age distribution of health levels of male and female migrant workers, confirms the phenomenon of health inequality in migrant workers, and explores the possible causes of health inequality from the perspective of education level and insurance purchase.

## Methods

### Data Source

In this study, we used data from the China Migrants Dynamic Survey (CMDS) conducted by the National Health Commission in China in 2018. The CMDS is a large-scale national sample survey of migrants that covers the concentration of migrants in 31 provinces, autonomous regions, and municipalities. The survey covers migrants’ survival and development, migration characteristics, employment situation, income, living conditions, social integration, mental health, basic public, medical, and social insurance services, health status, and other areas. The survey sampling method employed the Probability Proportionate to Size Sampling (PPS) approach. In the first stage, villages and towns are selected according to the PPS method. In the second phase, villages were selected from these townships, following the PPS methodology. Finally, individual participants were randomly selected from these villages in the third phase.

### Variables

#### Dependent Variable

In this paper, we analyze the self-reported health status (SRH) of migrants as the key dependent variable. SRH is a subjective measurement method that combines biology, psychology, society, and function. Many studies have confirmed the reliability of SRH in measuring the overall health of individuals [17, 18]. Therefore, according to the CMDS in 2018, we use “How is your health status?” to indicate the overall assessment of migrants’ health. Responses were coded 1 for healthy and 0 for unhealthy.

#### Explanatory Variables

Age and gender are the core explanatory variables for this study. These factors would help us to analyze health disparities among migrant workers more effectively. Regarding gender, we assigned a value of 0 for males and 1 for females based on respondents’ responses. In terms of age, we calculated the specific age of the respondents as of May 2018, which was the time of the survey, based on their month and year of birth. At the same time, to understand the distribution of health differences among migrants over different ages, we grouped the samples by age into seven groups, with 10-year intervals ranging from 17 to 89 years old.

#### Control Variables

To obtain the impact of gender differences in migrant workers on health status, we controlled for other factors that may affect the health status of migrant workers, including personal and family characteristics. Personal characteristics include education, ethnicity, household registration system (Hukou), marital status, occupation, and industry type. Family characteristics include household income, etc. In addition, we further controlled for the inflow to the county fixed effect.

#### Education

We divided respondents into seven levels of education, specifically, no primary school = 1, primary school = 2, middle school = 3, high school/technical secondary school = 4, junior college = 5, undergraduate = 6, and postgraduate = 7.

#### Ethnicity

We divided the ethnicity of the migrant workers into Han and ethnic minorities, where Han is 0 and ethnic minorities are 1.

#### Occupation

We classified the occupations of the migrant workers by type of industry, primary industry = 1; secondary industry = 2; tertiary industry = 3.

#### Household Income

A significant positive relationship exists between income and health status [17, 18]. We controlled for the logarithm of migrant workers’ household income, a continuous variable.

#### Hukou

As an institution barrier, household registration system (*Hukou*) is excluded from the public resources in the residential areas, resulting in the division between urban and rural areas in providing public services such as education and medical care in China [19–23]. Therefore, we controlled for the dummy variable for the Hukou type (*rural Hukou* = 0, *others* = 1).

#### Marital Status

Marriage can bring social resources and relationships to individuals, improving health outcomes [24–26]. Therefore, We classified the marital status of the migrant workers as married and unmarried, with single, divorced, widowed, cohabitation considered unmarried, and first marriage or remarried considered married. This attribute was measured as a dummy variable (unmarried = 0, married = 1).

Descriptive statistics for all variables are shown in Supplementary File S1.

### Model Design

We construct a multiple linear regression model to study the gender differences of health status. The model is presented in Eq. 1 as follows:

$$Y_{i}=\beta_{1}age*{female}_{i}+\beta_{2}{age}_{i}+\beta_{3}{female}_{i}+X_{i}\gamma +\mu_{i}$$ (1)

Where i denotes individual, the dependent variable $Y_{i}$ represents the individual’s health status. β and γ are the parameters of the estimated value, and $\beta_{1}$ is the critical parameter of our analysis. It represents the health differences between different age groups. Age denotes the age group of individual i ; female represents the gender of individual i ; and X represents other control variables, including educational, ethnic, industry type, household income, Hukou, marital status, and regional fixed effects. The error term μ denotes the random error.

## Results

### Gender Differences in Health Status of the Migrant Workers

**FIGURE 1:**
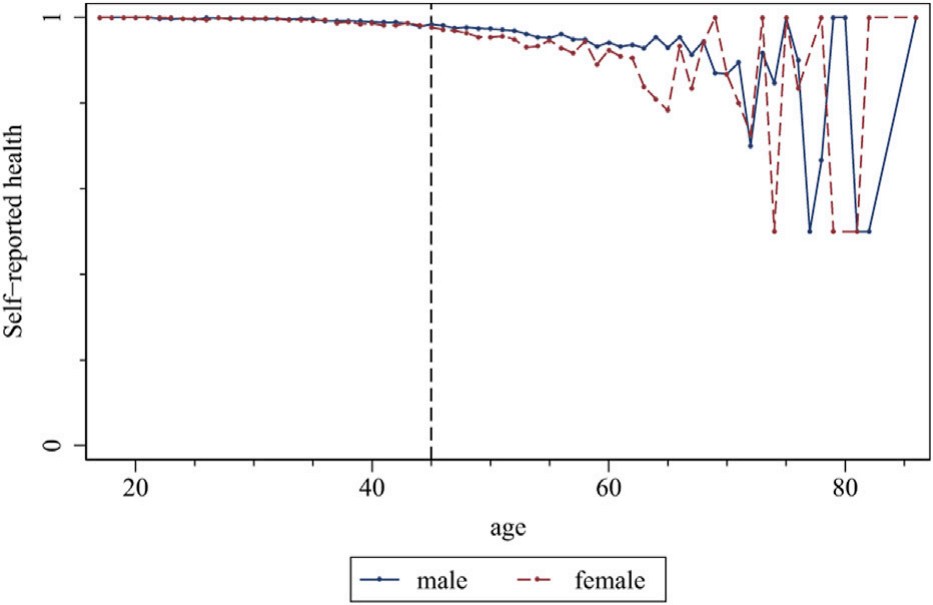
Self-reported health status of the migrant workers at different age (China. 2018).

In the first stage of the analysis, we present evidence in graph form. Figure 1 shows the study results, indicating that females generally reported worse health than males. Meanwhile, when we divide the migrant workers by age group, we find that the health status of both male and female groups follows the same trend: as age increases, health status declines, and this trend is more pronounced in females over 45.

In the regression estimation, we also classified the workers by age, with [27, 28] as the reference group. Table 1 shows the regression results. Each column presents the estimated results using Eq. 1 with or without controls.

**TABLE 1:** Self-reported health status of the migrant workers at different ages (China. 2018).

	(1)	(2)	(3)
Female	−0.0277*** (0.00238)	−0.0256*** (0.00234)	−0.0168*** (0.00236)
Female*age (16, 25)	0.0198*** (0.000902)	0.0186*** (0.000965)	0.0159*** (0.00108)
Female*age (25, 35)	0.0192*** (0.000626)	0.0182*** (0.000637)	0.0130*** (0.000616)
Female*age (35, 45)	0.00355*** (0.00108)	0.00321** (0.00107)	0.00572*** (0.00109)
Female*age (50, 60)	−0.0711*** (0.00329)	−0.0663*** (0.00321)	−0.0559*** (0.00323)
Female*age (60, 70)	−0.160*** (0.00784)	−0.149*** (0.00769)	−0.135*** (0.00770)
Female*age (70, 80)	−0.256*** (0.0169)	−0.241*** (0.0165)	−0.225*** (0.0164)
Female*age (80, 100)	−0.373*** (0.0445)	−0.354*** (0.0433)	−0.332*** (0.0434)
X	NO	NO	YES
City	NO	YES	YES
N	46,183	46,133	46,133

Notes: 1) Dependent variable: self-reported health status. 2) The control age group is (45, 50). 3) The control variables X include education, ethnicity, occupation, household income, Hukou, marital Status, and so on. City represents fixed effects after controlling for cities. 4) ***, **, * indicates statistical significance of 1%, 5% and 10% levels, respectively. Numbers in parentheses are standard errors for robust SE.

We begin the estimation by examining self-reported health, without controlling for any variables, in Column (1). Consistent with Figure 1, females have significantly lower health status than males. Specifically, after observing the coefficients of the interaction terms, we can find that the coefficients of the interaction terms have significant variations around the age group (45–50). Below this age group, the coefficients of interaction terms are positive, but the coefficients of interaction terms above this age group become negative, and the absolute values of these coefficients are expanding. This result indicates that the differences in health between females and males decrease before age 45. However, the differences in health status between females and males widen after that. Next, in Columns 2 and 3, we sequentially add relevant control variables and city-fixed effects. The results show that the key coefficients are similar to Column (1) regarding the magnitude and significance level, suggesting a negligible influence exerted by the omitted variables.

## Discussion

Based on the above analysis, we confirm significant gender differences in the health status of migrant workers in China: the health status of females is worse than that of males, which is consistent with the results of existing studies [29–31]. More importantly, we find that the health differences between females and males gradually decrease before age 45. However, after the age of 45, the differences in health status between females and males are expanding. In the next phase of the study, we will further discuss the possible causes of these health differences.

### Possible Reasons for Gender Differences in the Health Level of Migrant Workers

At present, the analysis of the health status of migrant workers and influencing factors mainly focuses on individual and socio-economic factors [32]. Compared to traditional medical and health factors, non-medical and health factors such as education level, family background, and living habits make a more significant marginal contribution in terms of personal factors. Education and health are the most closely related factors [33]. Research has shown that education can affect an individual’s health status in at least two ways. Firstly, the labor skills and knowledge acquired through education can be transformed into economic resources through productive capacity, which provides strong material support for an individual’s health [34, 35]. Secondly, education can improve healthy productivity, meaning highly educated individuals have stronger cognitive abilities and richer health knowledge and tend to choose a healthier lifestyle to maintain good health [36, 37].

One of the most critical social and economic factors that affect the health of migrant workers is the medical insurance system. Scholars are gradually paying more attention to the relationship between medical insurance and health status due to the rapid rise in medical costs. In this context, policymakers are focusing on health insurance’s input and output effects. Health insurance is considered a potential benefit for health, which has led to a great deal of research by scholars on the subject. Numerous studies from a global perspective, in both developing and developed countries, have shown that health insurance can improve an individual’s health status by increasing the utilization rate of health services [38–40].

### The Influence of the Educational Level of the Migrant Workers on the Health Status

**FIGURE 2:**
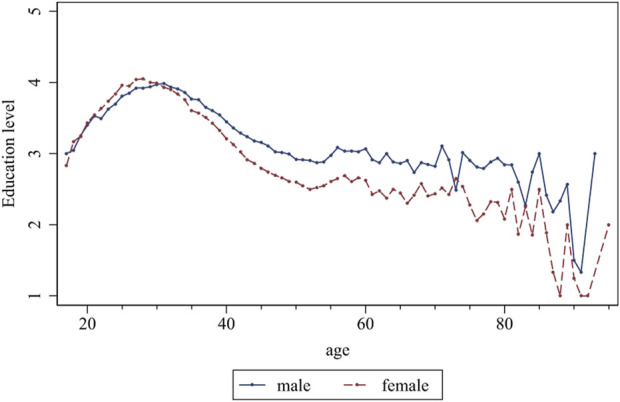
Educational level of the migrant workers of different genders (China. 2018).

To explore the impact of the educational level of migrant workers on their health, we analyzed the educational level of migrant workers in China using CMDS data from 2018. Figure 2 shows that there are significant gender differences in the educational level of migrant workers. Females have slightly higher levels of education than males in the young group; however, after the age of 30, the educational level of females gradually becomes lower than that of males, and the differences in education levels continue to expand with age.

At the same time, we further studied the differences in health status between males and females at different levels of education. The regression results (Table 2) indicated that the health status of females was significantly lower than that of males in groups with lower levels of education (junior middle school graduates and below). These differences were widening. Among the groups with high school graduation and above, although the health status of females was also lower than that of males, no trend exists of differences that increase with age. Furthermore, the gender gap in health status was not significant among older persons with higher levels of education (university graduates and above). The findings of Roxo’s study also indicated that poor health was more prevalent among women, and that women’s health disadvantage was more prominent in the less educated group, whereas the disparity in health between men and women was not significant in the group with high school education or above, which is consistent with our own results [41]. Solé-Auró et al. investigated the gender gap in unhealthy life expectancy across education levels and ages in Spain. They found that there were significant gender differences across most age groups, with women experiencing poorer health than men, and this gender difference was particularly pronounced among the low educated [42]. Similar results were observed by Di Lego et al. [43] in Austria and Vloo et al. [44] in Netherlands. This shows that educational level is an important factor affecting gender inequalities in health, which is in line with our findings.

**TABLE 2:** Self-rated health status of the migrant population under different educational levels (China. 2018).

	(1) Primary school graduates and below	(2) Junior middle school graduate	(3) Senior middle school graduate	(4) University graduates and above
Female	−0.0148** (0.00494)	−0.0137*** (0.00299)	−0.0104 (0.00565)	−0.0157* (0.00759)
Female*age (16, 25)	0.0539*** (0.00873)	0.0133*** (0.00151)	0.00737*** (0.00164)	0.00177 (0.00173)
Female*age (25, 35)	0.0438*** (0.00474)	0.0171*** (0.000920)	0.00986*** (0.00107)	0.00310*** (0.000749)
Female*age (35, 45)	0.0150*** (0.00407)	0.00388** (0.00149)	0.00568** (0.00181)	0.00145 (0.00135)
Female*age (50, 60)	−0.0563*** (0.00569)	−0.0478*** (0.00462)	−0.0425*** (0.00784)	−0.0412** (0.0159)
Female*age (60, 70)	−0.145*** (0.0117)	−0.0997*** (0.0136)	−0.0831*** (0.0162)	−0.0938*** (0.0278)
Female*age (70, 80)	−0.247*** (0.0227)	−0.194*** (0.0331)	−0.121*** (0.0358)	−0.0973* (0.0486)
Female*age (80, 100)	−0.318*** (0.0523)	−0.364*** (0.110)	−0.0498 (0.0549)	−0.199 (0.182)
X	YES	YES	YES	YES
City	YES	YES	YES	YES
N	28,944	74,213	37,216	29,488

Notes: 1) Dependent variable: self-rated health status. 2) The control age group is (45, 50). 3) The control variable X and fixed effects of cities have been added to all analyses. 4) ***, **, * indicates statistical significance of 1%, 5% and 10% levels, respectively. The numbers in parentheses are standard errors of robust SE.

### Gender Differences in Medical Insurance Participation Rate of Migrant Workers

At the end of 1990s, China began to reform the medical insurance system and introduced a new form of medical insurance: Urban Employee Basic Medical Insurance (UEBMI). However, the UEBMI is based on employment and linked to one’s work unit, which makes it more difficult for unemployed or informally employed individuals, especially females, to be covered by medical insurance. This limitation can affect their health status [45]. Therefore, based on the UEBMI reform in China, we explore the possible reasons for the gender differences in the health level of migrant workers from the perspective of whether they purchase medical insurance.

**FIGURE 3:**
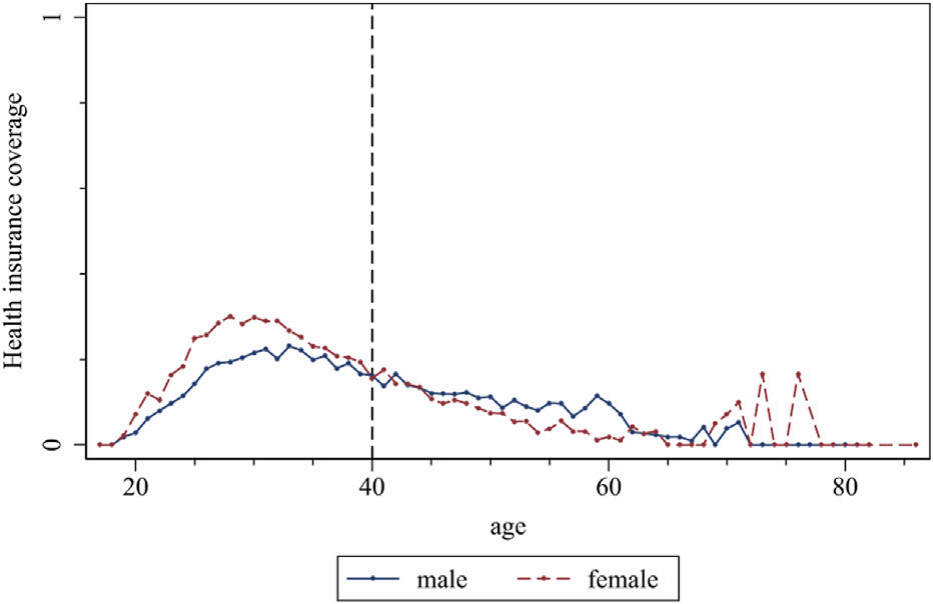
The Urban Employee Basic Medical Insurance Coverage Rate at different genders (China. 2018).

We used the CMDS in 2018 to analyze the participation of health insurance for migrant workers in China. Figure 3 shows that the health insurance coverage rate of the migrant workers showed an inverted-U curve with age. Before age 40, females had a higher health insurance coverage rate than males. However, after that, females had a lower coverage rate than males.

We further analyzed the differences in health insurance coverage rates among migrant workers using Eq. 1, where the critical dependent variable $Y_{i}$ represents the health insurance coverage of the individual. Table 3 shows the regression results. First, we estimated the model without controlling for any variables. The regression results shown in Column (1) indicate that females have lower health insurance coverage than males, and the differences in health insurance coverage between females and males increase with age. Next, in Column (2), we controlled for city-fixed effects, and the key coefficients did not significantly change in magnitude or significance level compared to Column (1). Finally, when we controlled for related variables in Column (3), we found that although the coverage of health insurance for migrant workers tends to rise with age, this trend is influenced by gender, with less of an increase for females than males.

**TABLE 3:** Health insurance coverage of the migrant workers at different ages (China. 2018).

	(1)	(2)	(3)
Female	−0.00978 (0.00527)	−0.00657 (0.00461)	0.0318*** (0.00458)
Female*age (16, 25)	0.0476*** (0.00778)	0.0343*** (0.00673)	−0.0570*** (0.00744)
Female*age (25, 35)	0.0834*** (0.000626)	0.0727*** (0.00519)	0.00586 (0.00517)
Female*age (35, 45)	0.140*** (0.00579)	0.119*** (0.00504)	0.0139** (0.00534)
Female*age (50, 60)	−0.0833*** (0.0103)	−0.0762*** (0.00656)	−0.0390*** (0.00701)
Female*age (60, 70)	0.0490*** (0.00607)	0.0392*** (0.00493)	0.000722 (0.00496)
Female*age (70, 80)	−0.104 (0.0875)	−0.0966** (0.0367)	−0.00930 (0.0368)
Female*age ([80, 100)	−0.0302*** (0.00739)	−0.0340*** (0.00518)	−0.0277*** (0.00527)
X	NO	NO	YES
City	NO	YES	YES
N	79,178	79,178	79,178

Notes: 1) Dependent variable: health insurance coverage. 2) The control age group is (45, 50). 3) The control variables X include education, ethnicity, occupation, household income, Hukou, marital Status, and so on. City represents fixed effects after controlling for cities. 4) ***, **, * indicates statistical significance of 1%, 5% and 10% levels, respectively. Numbers in parentheses are standard errors for robust SE.

In terms of gender differences in insurance, Zhou et al. discovered that due to the fact that health insurance predominantly targets individuals with formal employment, women’s reduced participation rates in the labor force create additional challenges for them to acquire health insurance. Additionally, the likelihood of being laid off or encountering difficulty in securing employment during health insurance reform is greater for older women, resulting in lower chances of accessing health insurance [46]. Allcock investigated the sociodemographic patterns of healthcare insurance coverage in Namibia. The results indicate that males have a higher health insurance coverage rate compared to females, and there is a positive correlation between age and health insurance coverage rate [27]. Mulenga found that private health insurance coverage in Zambia has generally been low for both women and men, with the probability of having health insurance increases with age for both men and women. In addition, Mulenga et al. [47] also note that health insurance coverage varies with gender, with coverage favouring men. These findings are similar to those of our study. In summary, our results suggests that migrant workers also have gender differences in health insurance coverage, and this gender gap in insurance coverage may be an important contributor to their health inequalities [48].

### Limitations and Strengths

One of the limitations of our study is the use of SRH as a measure of individuals’ health status. This approach is subjective and may introduce bias, as individuals with higher education tend to understand their health status better than those with lower education. It’s better to use objective health measures such as mortality, morbidity including blood pressure, BMI, hypertension, and chronic disease, and health-related behaviors. However, we do not have data on these objective indicators of migrant workers’ health. This is a direction for our future study. Additionally, our study only examines the impact of education and health insurance on the health outcomes of migrant workers of different genders and does not consider urban-rural educational differences. We will also work on it in the future. One strength of our study is that there is limited existing literature on health inequalities among migrant workers of different genders. Our research enhances the understanding of health inequalities among migrant workers by examining potential causes of such inequalities in relation to both education level and insurance coverage.

### Conclusion

This paper explains the gender differences in the health status of migrant workers and the possible reasons for the differences from the perspective of UEBMI reform. First of all, based on the CMDS in 2018, we found significant differences in the health status of migrant workers regarding gender, with the health level of the female group being lower than that of the male group. Gender differences in health are more pronounced in older age groups (over 45 years old) compared to younger age groups. Secondly, we explored the possible reasons for health inequalities of migrant workers from the two directions of education level and insurance purchase. We found that female migrant workers have lower education levels and health insurance coverage than males. Finally, we further discussed the impact of education level and health insurance on the health status of migrant workers through regression analysis. Overall, the study shows that significant gender differences exist in the educational level and health insurance coverage of migrant workers in China. These differences are essential reasons for the gender differences in their health status.

Based on the above research, this paper draws the following policy implications: First, given the irreplaceable role of education in improving the health status of migrant workers, it is necessary to effectively improve their education level by providing formal training, such as vocational and adult education. Additionally, strengthening health education for migrant workers and enhancing their health awareness and literacy is crucial. Second, in the process of promoting the reform of the universal medical insurance system, priority should be given to addressing the problem of unbalanced and inadequate development of health insurance, to further improve access conditions for health insurance, and to promote the equalization of basic public medical services. Confronting gender differences in the health status of migrant workers and incorporating gender awareness into the health insurance reform plan is essential. Moreover, the interconnection and complementarity between the UEBMI system, the Urban Resident Basic Medical Insurance (URBMI) system, and the New Rural Cooperative Medical Care (NRCMS) system should be promoted. This collaboration will establish and perfect the public health service system [28, 49, 50].
