# Supplementary material for: Gender Differences in Migrant Workers Health in China
Source: Int J Public Health. 2023 Aug 14;68:1605018. doi: 10.3389/ijph.2023.1605018 (PMC10467421; doi:10.3389/ijph.2023.1605018)
Supplement: Supplementary file 1 [file Table1.PDF]

Table 1: Descriptive statistics（China. 2018）

Variable	Obs	Mean	Std	Min	Max
Health	152,000	3.85	0.41	1	4
Gender	152,000	0.49	0.5	0	1
Age	152,000	36.83	11.2	16	89
Education	152,000	3.51	1.19	1	7
Ethnicity	152,000	0.09	0.28	0	1
Occupation	127,105	2.69	0.51	1	3
Household income	127,010	8.78	0.62	0	13.82
Hukou	127,105	0.31	0.46	0	1
Marital Status	127,105	0.80	0.40	0	1
